# Stability and Expression Levels of HLA-C on the Cell Membrane Modulate HIV-1 Infectivity

**DOI:** 10.1128/JVI.01711-17

**Published:** 2017-12-14

**Authors:** Francesca Parolini, Priscilla Biswas, Michela Serena, Francesca Sironi, Valentina Muraro, Elisabetta Guizzardi, Lucia Cazzoletti, Maria Teresa Scupoli, Davide Gibellini, Elisabetta Ugolotti, Roberto Biassoni, Alberto Beretta, Mauro Malnati, Maria Grazia Romanelli, Donato Zipeto

**Affiliations:** a Department of Neurosciences, Biomedicine and Movement Sciences, University of Verona, Verona, Italy; b Division of Immunology, Transplantation and Infectious Diseases, IRCCS Ospedale San Raffaele, Milan, Italy; c Service of Transfusion Medicine, AOUI, Verona, Italy; d Department of Diagnostics and Public Health, University of Verona, Verona, Italy; e University Laboratory of Medical Research, Verona, Italy; f IRCCS Institute Giannina Gaslini, Genoa, Italy; Emory University

**Keywords:** AIDS, HIV-1, HLA, HLA-C, infection, MHC-I, major histocompatibility complex

## Abstract

HLA-C expression is associated with a differential ability to control HIV-1 infection. Higher HLA-C levels may lead to better control of HIV-1 infection through both a higher efficiency of antigen presentation to cytotoxic T lymphocytes and the triggering of activating killer immunoglobulin-like receptors on NK cells, whereas lower levels may provide poor HIV-1 control and rapid progression to AIDS. We characterized the relative amounts of HLA-C heterotrimers (heavy chain/β_2_ microglobulin [β_2_m]/peptide) and HLA-C free heavy chains on peripheral blood mononuclear cells (PBMCs) from healthy blood donors harboring both alleles with stable or unstable binding to β_2_m/peptide. We analyzed the stability of HLA-C heterotrimers of different allotypes and the infectivity of HIV-1 virions produced by PBMCs with various allotypes. We observed significant differences in HLA-C heterotrimer stability and in expression levels. We found that R5 HIV-1 virions produced by PBMCs harboring unstable HLA-C alleles were more infectious than those produced by PBMCs carrying the stable variants. We propose that HIV-1 infectivity might depend both on the amounts of HLA-C molecules and on their stability as trimeric complex. According to this model, individuals with low-expression HLA-C alleles and unstable binding to β_2_m/peptide might have worse control of HIV-1 infection and an intrinsically higher capacity to support viral replication.

**IMPORTANCE** Following HIV-1 infection, some people advance rapidly to AIDS while others have slow disease progression. HLA-C, a molecule involved in immunity, is a key determinant of HIV-1 control. Here we reveal how HLA-C variants contribute to the modulation of viral infectivity. HLA-C is present on the cell surface in two different conformations. The immunologically active conformation is part of a complex that includes β_2_ microglobulin/peptide; the other conformation is not bound to β_2_ microglobulin/peptide and can associate with HIV-1, increasing its infectivity. Individuals with HLA-C variants with a predominance of immunologically active conformations would display stronger immunity to HIV-1, reduced viral infectivity and effective control of HIV-1 infection, while subjects with HLA-C variants that easily dissociate from β_2_ microglobulin/peptide would have a reduced immunological response to HIV-1 and produce more infectious virions. This study provides new information that could be useful in the design of novel vaccine strategies and therapeutic approaches to HIV-1.

## INTRODUCTION

Several host determinants influence HIV-1 replication and immune responsiveness, resulting in variable disease progression. Genetic variants of the major histocompatibility complex class I (MHC-I) genes represent critical determinants associated with HIV-1 disease progression (1).

The MHC-I complex consists in a 45-kDa heavy α chain noncovalently associated with a light β chain called β_2_ microglobulin (β_2_m) and a short (8- to 11-mer) antigenic peptide. In humans, the main α chains are encoded by the HLA-A, -B, and -C loci. The role of MHC-I is to present foreign peptides to CD8^+^ T cells, which recognize the infected cells, and to control natural killer (NK) cell responses via interaction with killer immunoglobulin-like receptors (KIRs) leading to inhibition or activation of their cytolytic function (2).

HLA-C expression on the cell membrane is lower than that of HLA-A and -B (3, 4). Several mechanisms are involved in the regulation of HLA-C cell surface expression. (i) HLA-C itself does not efficiently associate with β_2_m (3) but, more importantly, shows more selective peptide binding characterized by a lower affinity constant than that of HLA-A and -B (5). (ii) HLA-C has a dihydrophobic motif (L336-I337) in the cytoplasmic tail that causes more rapid internalization than HLA-A and -B (6, 7). (iii) A subset of HLA-C allotypes presents in the 3′ untranslated region (UTR) of its mRNA a target sequence for a microRNA (miR-148a) able to interfere at the posttranscriptional level with HLA-C gene expression (8). Vince et al. recently reported a new single nucleotide polymorphism (SNP), rs2395471, located in the HLA-C promoter region, associated with variable HLA-C expression (9).

In addition to CD4, MHC-I molecules are also downregulated during HIV-1 infection. Specifically, HIV-1 Nef is responsible for the internalization of HLA-A and -B (10–12), while a recent study reported that Vpu downregulates HLA-C in primary HIV-1 isolates (13).

During the process of budding from the cell membrane, HLA-C molecules are incorporated into nascent HIV-1 virions together with other cell proteins, resulting in a higher number of MHC-I molecules than HIV-1 Env trimers (14, 15).

MHC-I-negative cell lines are nonpermissive for the replication of primary HIV-1 isolates, but HLA-C transfection in these cells rescues HIV-1 replication competence (16). Moreover, fusion efficiency is reduced in HLA-C-negative cells (17) and viruses produced in HLA-C-silenced cells are significantly less infectious than those produced in HLA-C-expressing cells (18). Further studies demonstrated that HLA-C is selectively incorporated into HIV-1 virions, associates with HIV-1 Env, and modulates viral infectivity (19). We recently reported that the interaction between HIV-1 Env and HLA-C selectively involves HLA-C free heavy chains present at the cell surface (20).

A genome-wide association study identified a SNP located 35 kb upstream of the HLA-C coding region (rs9264942), associated with differences in HLA-C expression levels, as a major genetic determinant of HIV-1 host control (1).

Subsequently, the causal variant responsible for these associations was identified in a SNP (rs67384697) that maps in the 3′ UTR mRNA of HLA-C alleles affecting the binding of the microRNA Hsa-miR-148a (8). This SNP was shown to partially influence cell surface expression of HLA-C with poorly expressing alleles that maintain an intact miR-148a binding site (263G) and highly expressing alleles in which the site is deleted (263del), thus escaping the regulation by interference exerted by that microRNA (8). Increased HLA-C expression was associated with delayed progression to AIDS in both African and European Americans, regardless of their distinct HLA-C frequencies and linkage relationships with HLA-B and -A (21).

Adding complexity to this matter, other studies failed to confirm the association between HLA-C expression and these genetic markers (22–25). Furthermore, a study of a very specific population in Nairobi, Kenya (26), found that the same HLA-C allotype, HLA-C*07, could be associated with either a low (HLA-C*07:01) or an increased (HLA-C*07:02) rate of seroconversion.

It has been hypothesized that higher HLA-C expression levels might trigger HIV-1-specific responses by increasing antigen presentation to cytotoxic T lymphocytes (CTLs) (21), binding to KIRs on NK cells, or a combination of these mechanisms, explaining the slower progression to AIDS (27).

The protective role of highly expressed HLA-C alleles in HIV-1 infection is in apparent contrast to our previous studies suggesting a role for the HLA-C–Env association in the enhancement of viral infectivity. We postulate here that this apparent contradiction may stem from the presence of different HLA-C conformations (20, 28).

According to this hypothesis, differences among HLA-C allotypes in the modulation of HIV-1 infectivity could rely on intrinsic differences in their binding stability as heterotrimeric complexes. As a consequence, some HLA-C alleles might display a higher dissociation rate than others, increasing the relative proportion of HLA-C free heavy chains on the cell surface available for interaction with HIV-1 Env.

By using pulse-chase experiments, Sibilio et al. (28) studied the stability of HLA-C assembly of eight serologically defined alleles, showing that HLA-C*06, -*05, -*08, and -*02 displayed greater stability than HLA-C*01, -*03, -*04, and -*07. Thus, to test our working hypothesis, we defined the first as stable and the latter as unstable HLA-C variants. In addition, since by neighbor-joining relationship analysis, HLA-C*12 and -*16 cluster with HLA-C*06, whereas HLA-C*15 clusters with HLA-C*02 and HLA-C*14 clusters with HLA-C*01 (29), we classified HLA-C*12, -*15, and -*16 as stable variants and HLA-C*14 as an unstable HLA-C variant.

Recently, Kaur et al. (30) reported that variations in the HLA-C α1/α2 domains influence the structure of the peptide-binding cleft and drive differential expression of HLA-C allomorphs at the cell surface. They observed that HLA-C*05 binds a larger range of peptides than HLA-C*07, thus stabilizing the trimeric complex on the cell surface. These results further support our hypothesis and the rationale for our classification, since HLA-C*05 and -*07 are, respectively, classified as stable and unstable in the present work.

We reported that HIV-1 infection increases HLA-C free chains on the cell surface (20). HLA-C alleles with unstable binding to β_2_m/peptide might generate free chains able to interact with HIV-1 Env, thus increasing viral infectivity.

The primary objective of the present study was to explore whether HLA-C alleles are not only differently expressed but also show different stabilities as trimeric complexes. To this aim, we compared the reactivities of two monoclonal antibodies (MAbs), DT9 and L31, specific for HLA-C/β_2_m/peptide heterotrimers and HLA-C free chains, respectively. The secondary objective was to preliminarily test *in vitro* whether HIV-1 infectivity might be influenced by HLA-C stability by using prototype R5- and X4-tropic viruses.

## RESULTS

### Study population.

PBMCs were collected from 500 healthy bone marrow donors enrolled at the Italian Bone Marrow Donor Registry (IBMDR) and followed by the Service of Transfusion Medicine (AOUI, Verona, Italy). They were typed for HLA-A, -B, and -C by high-resolution molecular biology methods.

Since different HLA-C allotypes may differ in the stability of β_2_m/peptide binding and may be expressed at different levels, subjects with one stable and one unstable HLA-C allele were excluded because they are expected to show intermediate phenotypes. To highlight differences between the two allotype groups, we selected donors harboring both HLA-C alleles belonging to either the stable or the unstable group.

In addition to HLA-C, DT9 and L31 recognize some HLA-B alleles (28, 29, 31, 32). Thus, subjects expressing DT9 (HLA-B*13:01, -*35:01, -*40:06, and -*73:01)- and L31 (HLA-B*07, -*08, -*22, -*35, -*46, -*51, -*54, and -*56)-cross-reactive HLA-B allotypes were also excluded. According to these criteria, only about 10% of the potential donors were suitable for this study (Table 1).

**TABLE 1:** Summary of the population studied

Donor	Sex^*a*^	Age (yr)	Alleles			Stability group^*b*^
			HLA-A	HLA-B	HLA-C	
1	M	19	*02:01, *30:01	*40:01, *58:01	*03:02, *03:04	U
2	M	37	*11, *68	*37, *44	*06, *16	S
3	M	31	*02, *29	*18, *58	*07, *07	U
4	M	35	*02, *33	*14, *44	*05, *08	S
5	M	29	*02, *24	*44, *58	*07, *07	U
6	F	20	*02:01, *03:01	*27:05, *44:02	*02:02, *05:01	S
7	M	36	*02, *26	*15, *55	*03, *03	U
8	M	18	*11:01, *29:02	*39:01, *44:03	*12:03, *16:01	S
9	F	21	*25:01, *68:02	*39:01, *45:01	*12:03, *16:01	S
10	F	26	*02, *02	*15, *15	*04, *04	U
11	M	41	*24:02, *25:01	*18:01, *44:03	*12:03, *16:01	S
12	M	24	*02, *02	*44, *49	*05, *15	S
13	M	27	*02:01, *24:02	*38:01, *44:02	*05:01, *12:03	S
14	M	25	*02:01, *11:01	*38:01, *44:03	*12:03, *16:01	S
15	M	47	*02:01, *02:01	*44:05, *44:05	*02:02, *02:02	S
16	M	27	*02:01, *26:01	*15:01, *49:01	*04:01, *07:01	U
17	M	25	*23, *32	*15, *44	*01, *04	U
18	M	25	*02:01, *03:01	*15:01, *18:01	*03:03, *07:01	U
19	F	33	*26, *03	*13, *44	*05, *06	S
20	M	29	*11:01, *30:04	*44:02, *50:01	*06:02, *16:04	S
21	F	22	*02, *33	*14, *39	*08, *12	S
22	M	42	*03, *24	*39, *44	*04, *04	U
23	M	38	*02:01, *26:01	*18:01, *38:01	*12:03, *12:03	S
24	F	23	*11, *23	*18, *44	*04, *07	U
25	M	38	*26, *26	*37, *37	*06, *06	S
26	M	31	*01:01, *26:01	*38:01, *57:01	*06:02, *12:03	S
27	F	30	*02:01, *03:01	*13:02, *50:01	*06:02, *06:02	S
28	M	42	*02:01, *30:02	*18:01, *49:01	*05:01, *06:02	S
29	M	47	*31:01, *03	*18:01, *40:01	*03:04, *07:01	U
30	M	30	*30:02, *68:01	*18:01, *44:02	*02:02, *05:01	S
31	M	19	*01:01, *32:01	*14:01, *57:01	*06:02, *08:02	S
32	M	27	*02:01, *26:01	*18:01, *58:01	*07:01, *07:01	U
33	M	32	*33:01, *34:02	*14:02, *14:02	*08:02, *08:02	S
34	M	26	*03:01, *29:02	*39:06, *58:01	*07:01, *07:02	U
35	F	24	*01:01, *01:01	*37:01, *57:01	*06:02, *06:02	S
36	M	27	*01:01, *32:01	*41:02, *49:01	*07:01, *07:03	U
37	F	33	*01:01, *24:03	*18:01, *18:01	*07:01, *07:01	U
38	M	22	*02:01, *03:01	*38:01, *44:02	*05:01, *12:03	S
39	M	32	*01:01, *26:01	*37:01, *38:01	*06:02, *12:03	S
40	M	43	*11:01, *25:01	*18:01, *52:01	*12:02, *12:03	S
41	F	27	*25:01, *33:01	*14:02, *44:02	*05:01, *08:02	S
42	F	27	*03:01, *25:01	*13:02, *18:01	*06:02, *12:03	S
43	M	35	*02:01, *02:01	*18:01, *44:02	*07:01, *07:04	U
44	M	33	*03:01, *11:01	*14:01, *18:01	*05:01, *08:02	S
45	F	53	*24:02, *32:01	*37, *44	*01:02, *04:10	U
46	F	54	*11:01, *23:01	*44:02, *38:02	*04:01, *07:02	U
47	M	53	NA^*c*^	*49, *58	*07, *07	U
48	M	40	NA	*15, *49	*07, *07	U
49	M	50	*02:01, *29:01	*18, *44	*08:09, *16:01	S
50	M	39	*24:02, *30:11	*13:02, *37:01	*06, *06	S
51	M	43	NA	*37, *44	*06, *16	S
52	M	57	NA	*38, *52	*12, *12	S
53	M	45	*01:01, *02:05	*15:03, *49:01	*06, *12	S

a M, male; F, female.

b S, stable; U, unstable.

c NA, not available.

The groups considered were homogeneous and did not show any difference in gender (25/33 males in the stable group, 15/20 males in the unstable group; χ^2^ test, *P* = 0.9505) or age (mean age, 32.76 ± 1.69 years in the stable group and 33.90 ± 2.41 years in the unstable group; *t* test, *P* = 0.6919).

We compared the frequency of HLA-C alleles in the donors selected for this study with both the frequency of HLA-C alleles in the registered IBMDR donors and the frequency of HLA-C alleles reported in northern Italy (33). We observed some differences (Fisher exact test) between the selected population and the whole IBMDR population, in particular, an increase in HLA-C*06 (*P* = 0.0356), -*08 (*P* = 0.0299), and -*12 (*P* = 0.0412) frequencies and a decrease in HLA-C*04 (*P* = 0.0063) and -*15 (*P* = 0.0291) frequencies in the selected population. These differences are most likely due to the exclusion from the study of donors with cross-reactive HLA-B alleles which are in linkage disequilibrium with specific HLA-C alleles, since haplotypes tend to be inherited together as a haplotype block. Some common haplotypes in the Italian population are indeed reported to be HLA-B*35:01–HLA-C*04:01 and HLA-B*51:01–HLA-C*15:02 (Allele Frequency Net Database [AFND]; http://www.allelefrequencies.net) (34).

Exclusion of cross-reactive HLA-B alleles is necessary; this might have been a serious flaw in previous studies. In addition, MAb DT9 has been reported to be cross-reactive with HLA-E (35, 36). Lo Monaco et al. reported some concerns about this cross-reactivity, which may lead to overestimation of the presence of HLA-C on the cell surface (37). A later study (38), although conducted with a specific HLA-A, -B, and -C haplotype, reported that HLA-E is expressed at an about 25 times lower level that HLA-C, thus reducing concerns about the significance of MAb DT9 HLA-E cross-reactivity.

### Flow cytometry analysis of different HLA-C conformations on the cell surface.

**FIG 1:**
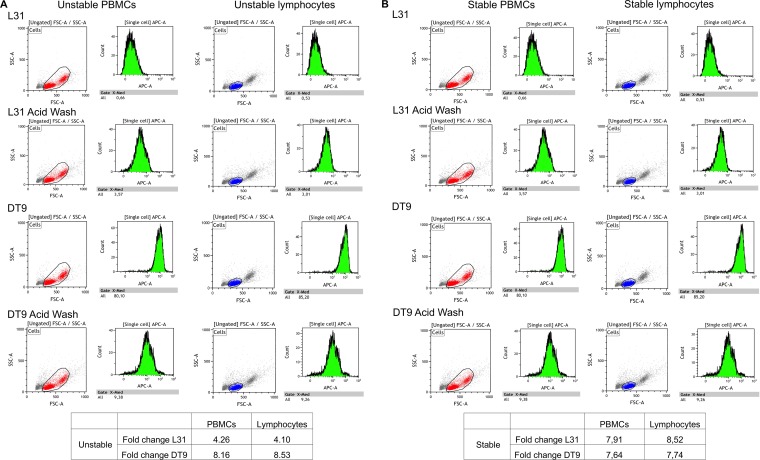
Flow cytometry analyses of HLA-C/β_2_m/peptide heterotrimers and HLA-C free chains. Gating strategies and representative histograms for the flow cytometry experiments are shown. MAb DT9 recognizes heterotrimers; MAb L31 recognizes HLA-C free chains. An acid wash was performed to remove β_2_m/peptide from HLA-C heterotrimers. Results for a subject with both unstable HLA-C alleles (A) and a subject with both stable HLA-C alleles (B) are shown. Fold change was calculated as the ratio of RMFIs after and prior to the acid wash for MAb L31 and as the ratio of RMFIs prior to and after the acid wash for MAb DT9. RMFIs were calculated as reported in Materials and Methods. MFI_control_ (secondary antibody control) was 0.19 or 0.26 for unstable PBMCs, 0.15 or 0.20 for unstable lymphocytes, 0.17 or 0.15 for stable PBMCs, and 0.12 or 0.10 for stable lymphocytes, respectively, without or with previous acid wash treatment. Fold change analyses of PBMCs and lymphocytes from the same donor show similar results for both antibodies. FSC, forward scatter; SSC, side scatter.

Preliminary control experiments showed no relevant differences in HLA-C stability between PBMCs and lymphocytes measured by analyzing L31 and DT9 reactivity. A representative comparison of PBMCs and lymphocytes of donors harboring unstable and stable HLA-C variants is reported in Fig. 1A and B, respectively. Thus, the following analyses were performed with PBMCs, as these were used for Western blotting and infectivity experiments.

**FIG 2:**
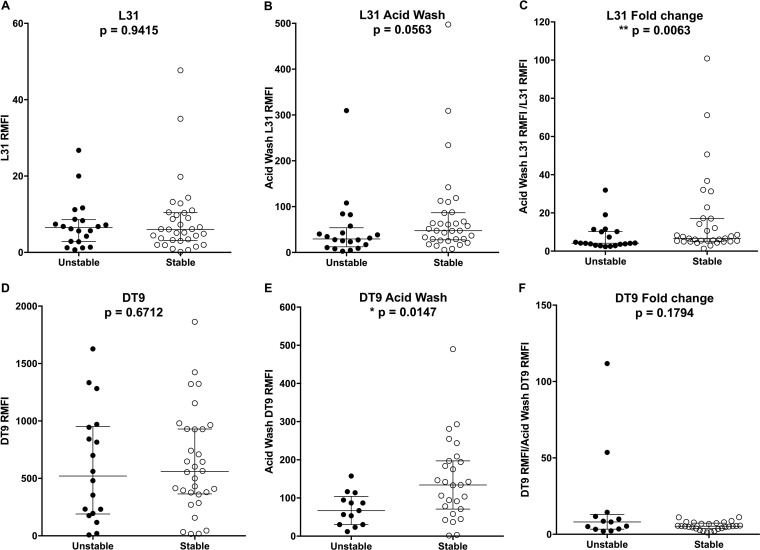
MAb L31 and DT9 flow cytometry analyses of HLA-C conformations expressed on the plasma membrane of PBMCs. Results obtained with cells from selected donors harboring unstable (black dots) or stable (white dots) HLA-C variants are displayed. Horizontal bars indicate medians and quartiles. Comparisons (Wilcoxon test) of the two groups: A, MAb L31, specific for HLA-C free chains, unstable *n* = 20, stable *n* = 33; B, MAb L31, after an acid wash to remove β_2_m, unstable *n* = 20, stable *n* = 33; C, L31 RMFI fold change in fluorescence, expressed as the ratio of the L31 RMFI after an acid wash to the L31 RMFI prior to the acid wash, unstable *n* = 20, stable *n* = 33; D, MAb DT9, specific for properly folded HLA-C heterotrimers, unstable *n* = 18, stable *n* = 32; E, MAb DT9, after an acid wash to remove β_2_m, unstable *n* = 13, stable *n* = 27; F, L31 RMFI fold change in fluorescence, expressed as the ratio of the DT9 RMFI prior to an acid wash to the DT9 RMFI after an acid wash, unstable *n* = 13, stable *n* = 27.

Labeling with MAb L31 showed comparable levels of HLA-C free chains in the stable and unstable groups (Fig. 2A; Wilcoxon test, *P* = 0.9415).

Similarly, the DT9 MAb (which recognizes the HLA-C/β_2_m/peptide complex) did not reveal significant differences between the two groups (Fig. 2D; Wilcoxon test, *P* = 0.6712).

A marginal difference was observed when the L31 analysis was performed after acid treatment (acid wash), which disrupts the majority of trimeric HLA-C complexes, allowing the quantification of a larger fraction of HLA-C free chains present on the cell surface (Fig. 2B; Wilcoxon test, *P* = 0.0563). As expected, the relative median fluorescence intensity (RMFI) of L31 staining after the acid wash was higher than the RMFI of constitutive L31, as visible by the different scale (Fig. 2A and B). Vice versa, as expected, the RMFI of DT9 staining after the acid wash was lower than the RMFI of constitutive DT9 (Fig. 2D and E). However, DT9 staining after the acid wash was significantly higher in the stable group (Fig. 2E; Wilcoxon test, *P* = 0.0147), underlining the presence of a larger pool of stable HLA-C trimers on these cells.

### HLA-C/β_2_m/peptide binding stability analysis.

HLA-C/β_2_m/peptide binding stability was measured as the fold change in fluorescence calculated as the ratio of L31 MAb reactivities after and before acid wash treatment. This value represents the proportion of HLA-C free heavy chains released after the disruption of the trimeric complexes and those constitutively present on the cell surface as a consequence of the natural instability of the HLA-C/β_2_m/peptide complexes. If the proportion of HLA-C in its free heavy chain conformation is greater than that of HLA-C heterotrimers, this ratio will be marginally influenced by the acid wash treatment and the L31 fold change in fluorescence will be relatively low. Vice versa, if HLA-C molecules are present mainly as heterotrimers, the acid wash treatment will have a greater effect in switching the HLA-C conformation and consequently the L31 fold change ratio will be higher.

Our hypothesis was that HLA-C allotypes classified as stable are more stably associated with β_2_m/peptide and consequently present a smaller proportion of free heavy chains than unstable allotypes. Therefore, larger and smaller fold increases in fluorescence should be expected for the stable and unstable allotypes, respectively.

Indeed, flow cytometric analyses indicated that cells from subjects in the unstable group have a significantly smaller fold change in fluorescence than those from subjects in the stable group (Fig. 2C; Wilcoxon test, *P* = 0.0063). We did not observe the prevalence of certain HLA-C allotypes in those subjects showing the greatest fold change in fluorescence, excluding the involvement of specific HLA-C variants. On the contrary, no difference was observed when the DT9 RMFI fold change was analyzed (Fig. 2F; Wilcoxon test, *P* = 0.1794).

**FIG 3:**
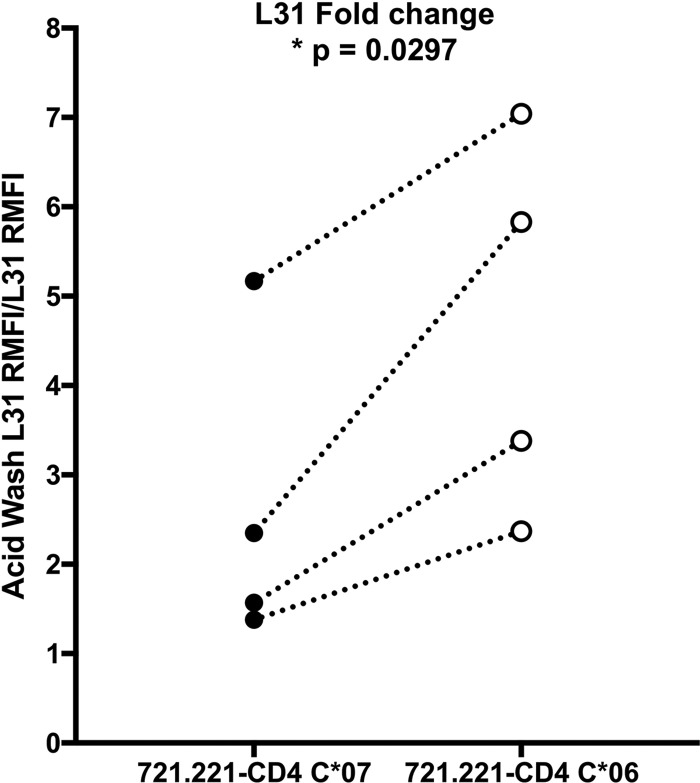
Analysis of fold changes in fluorescence in 721.221-CD4 cells. L31 RMFI fold changes in fluorescence in HLA-C*07 (unstable, black dots)- and -*06 (stable, white dots)-expressing 721.221-CD4 cells are shown. The graph represents the flow cytometry results of four independent transfections; the dotted lines connect results of the same transfection experiments. Two-way ANOVA was applied (fold change as the dependent variable, HLA-C allele and experiment replicas as independent variables).

The difference in the L31 RMFI fold change observed in PBMCs was further confirmed in a cell line model. We used 721.221-CD4 cells, which harbor a deletion of the MHC-I locus, transfected with either the HLA-C*06 (stable) or the HLA-C*07 (unstable) gene. Flow cytometry analysis with MAb L31, with or without an acid wash, revealed a greater fold change in fluorescence in HLA-C*06-expressing cells than in HLA-C*07-expressing cells in four independently replicated experiments (Fig. 3; two-way analysis of variance [ANOVA], *P* = 0.0297). This result confirmed the data obtained with PBMCs indicating that HLA-C*06 has a greater stability of the trimeric complexes than HLA-C*07. This experiment, performed in a controlled cellular model, excluded any other variable due to individual differences between subjects or to immune system involvement.

### Different total HLA-C expression levels in stable and unstable groups.

**FIG 4:**
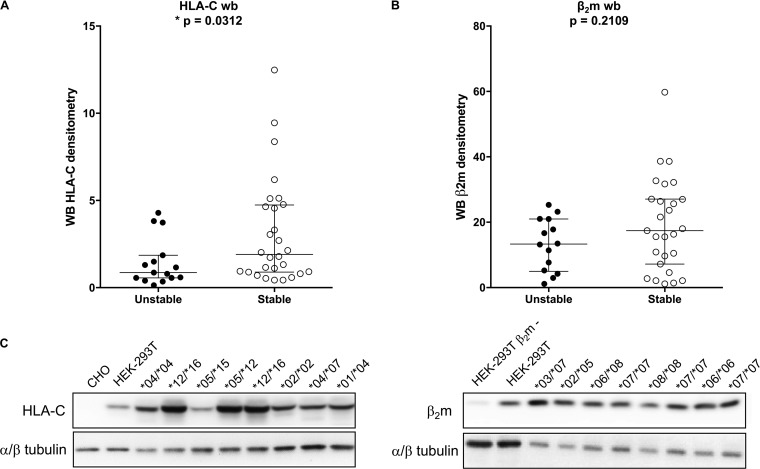
HLA-C and β_2_m Western blot analyses of PBMCs. Densitometric analyses of Western blot (WB or wb) assays for HLA-C (A) and β_2_m (B) for unstable (black dots) and stable (white dots) HLA-C allotypes are shown. Horizontal bars indicate medians and quartiles. For HLA-C (A), unstable *n* = 16, stable *n* = 28; Wilcoxon test; for β_2_m (B), unstable *n* = 14, stable *n* = 27; Wilcoxon test. (C) Representative images of Western blot experiments used to quantify HLA-C (left) and β_2_m (right) expression levels. Negative and positive controls are CHO and HEK-293T cell lysates for HLA-C and HEK-293T β_2_m-negative and HEK-293T cell lysates for β_2_m, respectively. The HLA-C allotype is depicted for the PBMC samples shown.

To test total HLA-C expression levels in our study population, we performed Western blot analyses of whole PBMC lysates. In addition, we assessed β_2_m expression levels. Significantly greater expression of HLA-C was observed in the stable group than in the unstable group (Fig. 4A; Wilcoxon test, *P* = 0.0312), whereas no significant differences in β_2_m expression levels were observed (Fig. 4B; Wilcoxon test, *P* = 0.2109). The significantly higher total HLA-C expression in the stable group might be a consequence of the higher expression of some HLA-C variants (i.e., HLA-C*02, -*06, and -*15) associated with SNPs rs67384697 (8) and rs2395471 (9) grouped as stable and the lower expression of variants (i.e., HLA-C*03 and -*07) grouped as unstable.

### HLA-C allotypes modulate the infectivity of HIV-1 virions.

**FIG 5:**
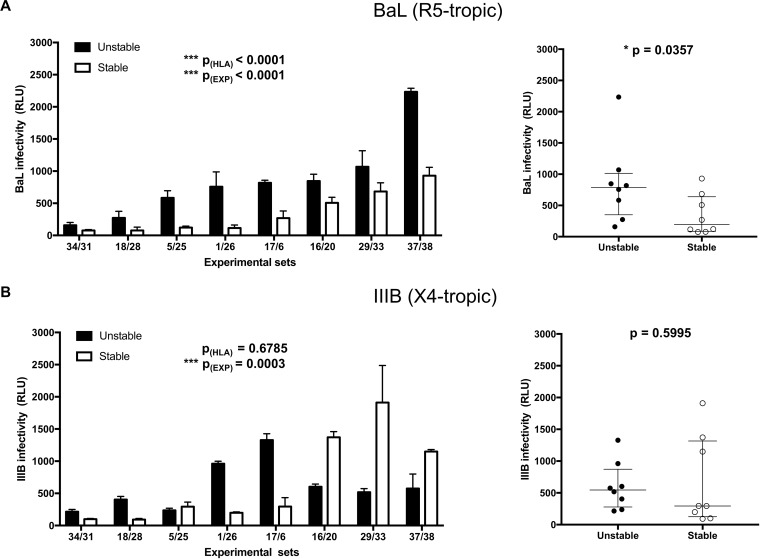
Infectivity of HIV-1 virions produced by PBMCs. Histogram charts represent the mean HIV-1 infectivity and standard deviations of different randomly coupled subjects harboring both stable (white bars) and unstable (black bars) HLA-C alleles. *x* axis, experimental set; *y* axis, virion infectivity expressed in RLU. Statistically significant differences were evaluated by two-way ANOVA. p_(HLA)_ refers to virion infectivity due to the HLA-C group (stable/unstable), while p_(EXP)_ refers to experimental set variability. (A) Infection conducted with the R5-tropic BaL HIV-1 strain. (B) Infection conducted with the X4-tropic IIIB HIV-1 strain. Dot charts represent the distributions of the mean infectivity of each subject (unstable HLA-C variants, black dots; stable variants, white dots). Horizontal bars indicate medians and quartiles; data were analyzed by Wilcoxon test.

The efficiency of infection of primary mixed cells such as PBMCs is quite variable because of several different factors (the numbers of CD4^+^ and CD8^+^ T lymphocytes, expression of HIV-1 coreceptors, phytohemagglutinin [PHA] activation, cell growth, cell viability); thus, we set up a standardized system to compare the influences of stable and unstable HLA-C alleles on infectivity. To this aim, PBMCs from a donor with stable HLA-C allotypes and a donor with unstable HLA-C allotypes were randomly coupled and evaluated in parallel in the same experimental set. PBMCs were infected with HIV-1, and supernatants were normalized for p24 content and used to infect TZM-bl cells (39), thus minimizing the variability of the target of infection. The TZM-bl cell line is stably transfected with a beta-galactosidase (β-Gal) and a luciferase plasmid under the control of the HIV-1 Tat promoter. Supernatants from HIV-1 BaL (R5 tropic, Fig. 5A)- and IIIB (X4 tropic, Fig. 5B)-infected PBMCs from 16 donors (8 with stable and 8 with unstable HLA-C alleles) were used to infect TZM-bl cells. To assess virion infectivity, three or four replicated input p24 concentrations were utilized. For each experimental set, the first viral concentration not showing an evident cytopathic effect on cell viability was used for data analysis. Data were analyzed by two-way ANOVA, untangling the effect of the variability between experimental sets and the effect of HLA-C stability on viral infectivity. Significantly higher infectivity [p_(HLA)_ < 0.0001] with the R5-tropic BaL HIV-1 isolate was observed in the unstable group than in the stable one (Fig. 5A, left). No significant difference was observed with the X4-tropic HIV-1 IIIB isolate [Fig. 5B, left, p_(HLA)_ = 0.6785]. As expected, significant differences, due to the experimental set, were observed [p_(EXP)_ < 0.0001 and p_(EXP)_ = 0.0003 for BaL and IIIB, respectively]. The two groups were also analyzed by comparing the distributions of the mean infectivity of each subject. The Wilcoxon test showed a significant difference between the two groups in BaL infectivity (*P* = 0.0357, Fig. 5A, right) but not in IIIB infectivity (*P* = 0.5995, Fig. 5B, right).

**FIG 6:**
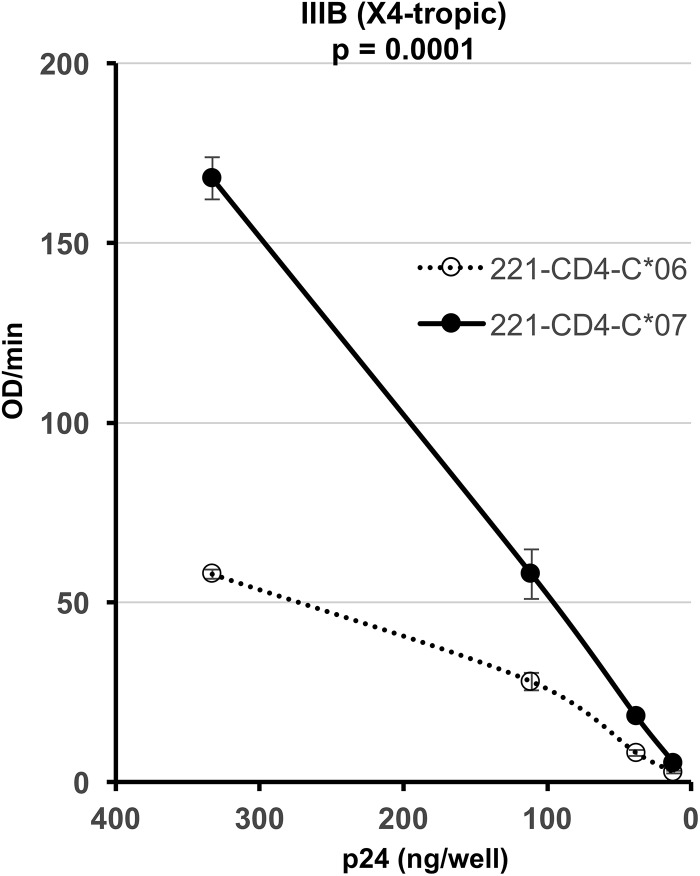
Infectivity of virions produced by 221-CD4-C*06 (stable variant) and 221-CD4-C*07 (unstable variant) cells. Four input p24 concentrations were used to infect TZM-bl cells in triplicate cultures. *x* axis, p24 pg/well; *y* axis, virion infectivity expressed in OD units/min. Black dots and continuous line, virions produced in 221-CD4-C*07 cells; white dots and dotted line, virions produced in 221-CD4-C*06 cells.

Finally, the infectivity of virions produced by 721.221-CD4 cells transfected with either HLA-C*06 or -*07 was assessed. Since 721.221-CD4 cells endogenously express CXCR4 but lack CCR5, they were infected with the HIV-1 IIIB isolate. The supernatants collected on day 3 were quantified for p24 content and used to infect TZM-bl cells. As shown in Fig. 6, the infectivity of virions produced by 721.221-CD4-C*07 cells was significantly greater than that of virions produced by 721.221-CD4-C*06 cells (two-way ANOVA, *P* = 0.0001).

## DISCUSSION

Here we studied whether HLA-C variants differ in the stability of β_2_m/peptide binding and whether the relative amounts of HLA-C/β_2_m/peptide complexes and HLA-C free heavy chains might be involved in the modulation of HIV-1 infectivity. Our working hypothesis predicts that a greater proportion of free heavy chains should be present on PBMCs obtained from individuals with unstable HLA-C alleles than on PBMCs obtained from donors with stable HLA-C alleles.

We analyzed the relationship between HLA-C associated with β_2_m/peptide and HLA-C free heavy chains (both as constitutive expression and after β_2_m/peptide stripping) on PBMCs, since the simple detection of only trimeric complexes, as reported previously (21, 24), might underestimate the actual amounts of HLA-C molecules that reach the cell surface (20).

When PBMCs from donors expressing both stable and unstable HLA-C allotypes were analyzed for HLA-C surface expression with either the DT9 (which recognizes HLA-C heterotrimers) or the L31 (which recognizes HLA-C free heavy chains) MAb, no significant differences were detected, suggesting that the overall amounts of HLA-C exposed at the cell membrane, although highly variable, are similar in the two groups. The necessary exclusion of DT9 and L31 HLA-B cross-reactive alleles from the study population, as well the different classifications of stable and unstable HLA-C alleles, might explain why we did not observe differences previously reported by others (21).

Of note, an acid wash treatment prior to flow cytometry dissociates the vast majority of HLA-C trimers, resulting in a sharp increase and reduction in L31 and DT9 reactivity, respectively. After the acid wash, higher, marginally significant reactivity was observed in the presence of stable alleles with MAb L31 and a higher degree of DT9-reactive molecules was displayed by the stable group, indicating that although the overall amount of HLA-C analyzed at the steady state on the cell surface was comparable in the two groups, differences in the composition of the HLA-C pools do exist.

This crucial aspect was further addressed by calculating the L31 fold change in the fluorescence ratio as an indicator of HLA-C/β_2_m/peptide binding stability. Hence, this value was significantly higher in the presence of stable HLA-C allotypes than in the presence of unstable ones. Furthermore, the fold change in fluorescence was analyzed in a cellular model, 721.221-CD4 cells, where single specific HLA molecules can replace the deletion of the MHC-I locus. 721.221-CD4 cells expressing HLA-C*06 (stable allele) presented a greater L31 fold change than cells expressing HLA-C*07 (unstable allele). The use of this cellular model, besides supporting our previous observations, excludes any other genetic or immunological components that may interfere in the experiments performed with PBMCs.

The significant difference in the fold change in fluorescence highlights a relevant molecular property of these interacting proteins, i.e., the stability of the heterotrimeric complexes that confirms our working hypothesis and defines the HLA-C*02, -*05, -*06, -*08, -*12, -*15, and -*16 allotypes as stable and the HLA-C*01, -*03, -*04, -*07, and possibly -*14 allotypes as unstable. Although all of the HLA-C alleles tested for assembly were characterized by a lower stability of the trimeric complexes than HLA-A or -B alleles (28), some alleles belonging to the unstable group (HLA-C*03 and -*07) showed the highest dissociation rate (28). Therefore, the presence of HLA-C on the cell membrane appears to be mediated by a combination of different important phenomena, i.e., HLA-C expression levels, depending on both HLA-C mRNA regulation mediated by miR-148a (21, 29) and, as more recently reported, by the SNP rs2395471 in the HLA-C promoter region (9), and the peptide affinity and stability of the HLA-C/β_2_m/peptide complexes, which, in combination, appear to determine not only the presence of different amounts of HLA-C on the cell membrane but also a different proportion of HLA-C full complexes and free heavy chains.

It is noteworthy that some low-expression HLA-C variants (9, 21), such as HLA-C*03 and -*07, are also the ones with lower β_2_m/peptide binding stability (28), while some stable HLA-C variants, such as HLA-C*02, -*06, -*12, and -*16, are also highly transcribed and expressed. This correlation is further highlighted by our observation, assessed by Western blot assay, of significantly lower HLA-C expression in the unstable group.

HLA-C*01 and -*04, highly expressed alleles according to Vince et al. (9), show lower β_2_m/peptide binding stability according to Sibilio et al. (28). Vince et al. did not report HLA-C mRNA expression for these alleles, while other studies (22–25) did not report any particular increase in their mRNA expression. Similarly, HLA-C*01 and -*04 were reported to have a high surface reactivity to MAb DT9 (21), but since they are also less stably bound to β_2_m/peptide, it is possible that DT9 staining underestimates the real amounts of HLA-C molecules on the cell surface. In all cases, the expression of HLA-C trimeric complexes on the cell surface depends on the integration of several factors such as mRNA levels, posttranslational regulation, transport, and differential stability of HLA-C alleles.

The proportion of HLA-C heterotrimers and free chains might play a role in the modulation of HIV-1 infection. We have shown that HIV-1 specifically increases the amount of free chains in cell lines either chronically or acutely infected (20). To study whether HIV-1 infection affected the levels and distribution of heterotrimers/free chains in PBMCs would have been a natural extension of our previous work, but here we chose to explore an aspect we deemed more important at first, which was whether the infectivity of HIV-1 virions is affected by HLA-C stability.

Indeed, the infectivity of virions produced in PBMCs of donors harboring unstable HLA-C alleles was significantly higher than the infectivity of virions produced by PBMCs of individuals carrying stable HLA-C alleles with the R5-tropic BaL HIV-1 isolate.

In addition, HIV-1 IIIB virions produced by 721.221-CD4-C*07 cells were more infectious than those produced by 721.221-CD4-C*06 cells. This experiment, performed in the same cellular background, confirms our hypothesis on HIV-1 infectivity and HLA-C stability. Vice versa, HIV-1 IIIB virions produced by PBMCs with unstable HLA-C alleles were not significantly more infectious than those produced by PBMCs with stable alleles.

According to this finding, a virion budding from PBMCs carrying unstable alleles might be more infectious because a greater proportion of HLA-C free chains would be available to interact with HIV-1 Env (20), determining an infectivity increase, as well as less susceptibility to neutralizing antibodies (16).

Fellay et al. (1) first described the correlation between HLA-C alleles and viral load variations during the asymptomatic set point period of infection, which is characterized by the predominance of R5 variants (40). Our data flow in the same direction, since the effect of unstable HLA-C alleles in enhancing viral infectivity is observed in PBMCs with an R5-tropic virus.

In a previous paper (18), we reported that the infectivity of some CXCR4-tropic isolates, namely, J500 and NDK, was not affected by the absence of HLA-C, suggesting a reduced effect of HLA-C on X4-tropic isolates. The effect of unstable HLA-C variants on the X4 HIV-1 isolate could be overshadowed by many other factors in the PBMC model, while it is evident in the 721.221-CD4 controlled model, in which the only difference is the expression of a stable (C*06) or unstable (C*07) variant.

The interplay between HLA-C and HIV-1 is a complex event, since HIV-1 requires the expression of HLA-C on the cell membrane to be more infectious (17–19), but at the same time, HLA-C participates in the activation of an appropriate CTL immune response (21, 41). The finding that HLA-C allelic variants have a different impact on viral infectivity *in vitro*, in the absence of any influence by the immune system, suggests that the availability of HLA-C free heavy chains is important in the modulation of HIV-1 infectivity. These data unravel a novel relationship between HLA-C expression and HIV-1, independent of cellular immunity.

Previous data published by our research groups (17–19) indicate that the presence of HLA-C, particularly as free heavy chains, positively modulates the infectivity of HIV-1 as a result of the association with HIV-1 Env protein at the cell membrane level (20). This effect may sum up and/or synergize with other determinants in defining the set point of individual HIV-1 infections.

The protective role of a higher expression level of HLA-C on the cell membrane appears to be in contrast to the increased infectivity of HIV-1 when HLA-C is incorporated into the virion. The results of this study may explain this apparent contradiction. Subjects with unstable HLA-C allotypes (HLA-C*01, -*03, -*04, -*07, and -*14) are characterized by the presence of a larger pool of HLA-C free heavy chains on the cell membrane, which in turn may increase viral infectivity. Conversely, subjects with stable HLA-C allotypes (HLA-C*02, -*05, -*06, -*08, -*12, -*15, and -*16) are characterized by the presence of a greater proportion of heterotrimers (HLA-C/β_2_m/peptide) on the cell membrane, which may stimulate cellular immunity and, consequently, determine better control of HIV-1 infection.

The present study, performed with a small population, owes its strength to the stringent selection carried out, which excluded all HLA-B allotypes cross-reactive with the DT9 and L31 MAbs and included only donors carrying both HLA-C alleles belonging to either the stable or the unstable group.

On the other hand, this same selection represents an extreme situation to test our working hypothesis. Certainly, in the general population, a continuous range of levels of susceptibility to HIV-1 infection would be expected because of the combination and interaction of different HLA-C allele expression levels, different stability as surface trimers, which follows a continuous rather than a bimodal pattern, and of many other genetic variants that may contribute to the outcome of the disease. For instance, an involvement of other HLA alleles could not be excluded. HLA-B*46, which is less stable according to Sibilio et al. (28), has been associated with susceptibility to HIV-1 infection (42). In the present study, we did not include subjects harboring HLA-B*46 because of its cross-reactivity with MAb L31.

Our results suggest that the stability of HLA-C complexes, in addition to the levels of HLA-C expression reported in previous studies, is involved in the modulation of HIV-1 infectivity. Their combination can influence the prediction of HIV-1 infection progression. In the asymptomatic, antiretroviral drug-untreated phase, individuals with low-expression and unstable HLA-C alleles might have a more rapid disease progression, whereas individuals with highly expressed and stable HLA-C alleles would better control HIV-1 infection. Expression levels alone do not explain why HIV-1 requires HLA-C on the cell membrane to be more infectious (16-18, 20) and, in addition, do not explain the higher serum and cerebrospinal fluid β_2_m concentrations observed in HIV-1-progressing patients (43–46), which may accumulate because of HLA-C trimeric complex instability.

The restricted availability of sample material from PBMC donors limited this study to testing of the infectivity of a reduced number of HIV-1 isolates. For the same reason, the correlation between SNPs rs2395471 and rs67384697 and the stability of each HLA-C allotype in PBMCs and purified CD4^+^ HIV-1-infected T cells could not be studied. These open questions deserve a new investigation.

HLA-C variants that may more easily dissociate will likely present a greater proportion of free heavy chains, increasing HIV-1 infectivity and promoting β_2_m release, also contributing to inflammatory states. It would be of interest to investigate whether HIV-1 Env facilitates the spontaneous decay of HLA-C trimeric complexes originating more HLA-C free chains available for Env interaction. Indeed, studies published in the early years of the HIV-1 pandemic reported that patients with HIV-1 dementia presented a higher concentration of β_2_m in their cerebrospinal fluid (45) and that β_2_m levels were raised in the serum of AIDS-related complex patients (44) and during HIV-1 infection progression (43, 46). Our results start to shed some light on these never fully explained observations; individuals with unstable HLA-C alleles might lose β_2_m, leading to its accumulation in their serum and cerebrospinal fluid. The role of β_2_m in neurodegenerative progression is not yet well defined, but it is known that β_2_m molecules can form fibrils (47, 48) and may be neurotoxic (49, 50).

Our present work underlines the significant role of HLA-C as a key player in the control of HIV-1 infection (51).

## MATERIALS AND METHODS

### Population and HLA and SNP rs67384697 genotyping.

The healthy blood donors involved in this study were enrolled at the IBMDR, followed by the Service of Transfusion Medicine, AOUI, Verona, Italy. This study was approved by the University of Verona Ethics Committee on 14 October 2015 (ProgCE 678CESC). All samples were collected after written informed consent was obtained from each donor.

The genetic analysis of 500 donors was performed with peripheral blood collected in Vacutainer tubes with EDTA as an anticoagulant. Samples were centrifuged at 2,000 rpm for 10 min at room temperature to obtain the middle ring enriched with leukocytes and platelets (buffy coat). The buffy coats were processed to extract the genomic DNA with the EZ1 Advanced XL kit (Qiagen S.r.l., Milan, Italy), and the extracted DNA was quantified by measuring the optical density at 260 nm (OD_260_) with a spectrophotometer.

Amplification was performed by sequence-specific oligonucleotide-primed reverse transcription-PCR (Luminex technology) with primers specific for the second and third exons of HLA-A, -B, and -C. The protocol was provided by Lagitre S.r.l., Milan, Italy. Data analysis was performed with the HLA Fusion Software (One Lambda).

Donors who were selected as suitable for this study had both HLA-C alleles belonging to either the stable group (HLA-C*02, -*05, -*06, -*08, -*12, -*15, and -*16) or the unstable group (HLA–*01, -*03, -*04, and -*07) and who did not have HLA-B alleles cross-reactive with the L31 and DT9 MAbs used (28, 29, 31, 32). Only about 10% of the donors typed satisfied these requirements.

The presence/absence of an intact miRNA binding site (rs67384697 G/deletion) was confirmed by a previously described pyrosequencing approach (27, 52) that allows direct analysis of the HLA-C 3′ UTR miR-148a binding site.

### PBMC purification.

Samples were obtained from donors selected according to the exclusion criteria. PBMCs were extracted from peripheral blood collected in Vacutainer tubes with the anticoagulant EDTA and Ficoll-Paque PLUS (GE Healthcare) in accordance with the manufacturer's protocol. Collected cells were aliquoted and stored in liquid nitrogen and subsequently used for flow cytometry and Western blot analyses within 2 weeks. Before use, the viability of cells consistently exceeded 90% in each sample, as assessed by trypan blue dye exclusion. Longer storage times were avoided to ensure reproducibility of data. When enough PBMCs were available, they were additionally used to test the ability to support HIV-1 infection.

### Flow cytometry.

MAb DT9, which recognizes a properly folded HLA-C/β_2_m/peptide heterotrimer (35), was a kind gift of A. Fenton-May and P. Borrow (Nuffield Department of Clinical Medicine, University of Oxford, Oxford, United Kingdom). MAb L31, specific for the α1 domain of HLA-C free heavy chain, not associated with β_2_m (32), was provided by P. Giacomini (Regina Elena Hospital, Rome, Italy). Preliminary experiments were conducted to compare the reactivity of PBMCs with that of lymphocytes, and no relevant differences were observed (Fig. 1).

To achieve the highest stripping of β_2_m and to preserve cell viability and integrity, a classical acid wash treatment was used to remove β_2_m (53, 54). This consisted of incubation of PBMCs in 0.3 M glycine HCl and 1% bovine serum albumin in RPMI (pH 2.5) for 3 min in ice, followed by a wash in RPMI to neutralize the pH and one in phosphate-buffered saline (PBS) prior to labeling with MAbs. PBMCs were surface labeled with either MAb DT9 (12.5 μg/ml) or L31 (1 μg/ml) for 40 min at 4°C. The cells were then washed with PBS and stained with allophycocyanin-conjugated goat anti-mouse antibody (BioLegend) for 30 min at 4°C. Cells were washed and analyzed with a FACSCanto flow cytometer. Dead cells and debris were excluded on the basis of forward and side scatter measurements, which confirmed a cell viability of >90% in each sample. Data were collected with the FACSDiva software (BD Biosciences), whereas data analysis was performed with the Kaluza software (Beckman Coulter).

The results obtained from staining with both MAbs DT9 and L31 were expressed as RMFIs calculated from median fluorescence intensity (MFI) values as follows: RMFI_sample_ = (MFI_sample_ − MFI_control_)/MFI_control_.

The fold change in the fluorescence ratio was calculated as the ratio of RMFIs after and prior to the acid wash for MAb L31 and between RMFIs prior to and after the acid wash for MAb DT9.

### Western blotting.

Protein extracts from PBMCs were spectrophotometrically quantified with Coomassie Plus Bradford Protein Assay Reagent (Thermo Scientific).

To evaluate the amount of HLA-C, 6 μg of protein was analyzed with MAb L31 (1 μg/ml). Protein lysates from HEK-293T and Chinese hamster ovary (CHO) cells were used as an internal standard and a negative control, respectively.

To evaluate the amount of β_2_m, 4 μg of protein was immunoblotted with a rabbit anti-β_2_m antibody (12 μg/ml; Abcam). HEK-293T and HEK-293T β_2_m-negative (20) cell lysates were used as an internal standard and a negative control, respectively.

The α/β-tubulin antibody was used as a loading control in accordance with the manufacturer's instructions (Cell Signaling). Horseradish peroxidase-conjugated anti-mouse (Promega) or anti-rabbit (PerkinElmer) IgG was used as a secondary antibody (0.5 μg/ml). The signal was developed with the ECL Advance Western blotting detection kit (Amersham) through the AutoChemi System UVP (BioImaging System).

The amounts of HLA-C and β_2_m were quantified through densitometric analysis with ImageJ software for OSX. Each sample value was normalized to its α/β-tubulin value. Differences between experiments were normalized with the same internal standard control (HEK-293T cell lysate).

### Cell lines.

CHO and HEK-293T cell lines (derived from human cervical adenocarcinoma) were obtained from the American Type Culture Collection (ATCC). β_2_m-negative HEK-293T cells were prepared with the CRISPR-Cas9 system as previously reported (20). The TZM-bl cell line (39) derived from HeLa (human cervix carcinoma) cells was provided by the EU Programme EVA Centre for AIDS Reagents, National Institute for Biological Standards and Control (ARP5011).

These cells were cultured in Dulbecco's modified Eagle's medium, high glucose (Euroclone), supplemented with 10% fetal bovine serum (FBS), 2 mM l-glutamine, 100 U of penicillin/ml, and 100 U of streptomycin/ml (Lonza). The 721.221-CD4 cell line, derived from B-lymphoblastoid cells not expressing HLA-A, -B, or -C because of the disruption of the HLA genetic locus (55), was a gift from A. Siccardi, San Raffaele Hospital, Milan, Italy. 721.221-CD4 cells were transfected with HLA-C*06- and -*07-expressing plasmids by nucleofection with the VCA-1003 kit (Lonza) by using the X-001 program in accordance with Amaxa's instructions. These cells were cultured in RPMI 1640 medium (EuroClone). The culture medium was supplemented with 10% FBS, 2 mM l-glutamine, 100 U of penicillin/ml, and 100 U of streptomycin/ml (Lonza).

All cell lines were grown at 37°C in a humidified atmosphere with 5% CO_2_ and routinely tested for the absence of mycoplasma contamination.

### Plasmids.

HLA-C*06 and -*07 sequences were obtained by retrotranscription of mRNA from HLA-C homozygous cell lines (MGAR and LBF, respectively) kindly provided by P. Giacomini (Regina Elena Hospital, Rome, Italy). Both sequences were cloned into plasmid pcDNA6.2 (Invitrogen) as previously described (20).

### Infection of PBMCs and TZM-bl cells.

The viability of all thawed cells, checked by trypan blue dye exclusion, was routinely about 95 to 98%. PBMCs were activated with PHA (5 μg/ml) for 48 h prior to overnight incubation with preparations of HIV-1 BaL (R5 or CCR5 user) and IIIB (X4 or CXCR4 user) with known titers (multiplicity of infection [MOI] range, 0.5 to 3) at 37°C. Excess virus was washed away, and PBMCs were plated at 10^5^/well and maintained in complete RPMI medium plus interleukin-2 (Proleukin; Novartis) at 200 U/ml.

PBMC culture supernatants were harvested at days 8 to 12 postinfection and frozen at −20°C until tested for p24 content. Supernatants from HIV-infected PBMCs were normalized for p24 content, and various input concentrations were used to infect TZM-bl. Triplicate cultures were set up, and infection was assessed after 2 days of culture.

### Infection of 721.221-CD4 and TZM-bl cells.

Both 721.221-CD4-C*06 and 721.221-CD4-C*07 cells were incubated overnight with preparations of the X4-tropic HIV-1 IIB isolate with known titers (MOI range, 0.5 to 3) at 37°C. 721.221-CD4 cell culture supernatants were harvested at day 3 postinfection and frozen at −20°C until tested for p24 content. Four input p24 concentrations were used to infect TZM-bl cells. Triplicate cultures were set up, and the experiment was repeated twice.

### Viral replication assay.

The HIV-1 structural protein p24 Gag was measured by a twin-site sandwich enzyme-linked immunosorbent assay (ELISA; Aalto Bio Reagents Ltd., Dublin, Ireland) based on a previously published method (56). Briefly, p24 antigen was captured from a detergent lysate of virions present in culture supernatants by a sheep polyclonal antibody adsorbed to a solid phase (3 h of incubation at room temperature). Bound p24 was detected with a mouse alkaline phosphatase-conjugated anti-p24 MAb (1 h of incubation) and a luminescence detection system. Luminescence was measured with a Mithras LB 940 luminometer (Berthold Technologies, Bad Wildbad, Germany) yielding relative luminescence units (RLU). By using a p24 internal standard curve, RLU were converted to ng/ml values. TZM-bl cells were lysed with 0.5% NP-40 (15 min at 37°C), and then 50 μl of lysate was transferred to a 96-well flat-bottom plate; this was followed by addition of the β-Gal substrate chlorophenol red-β-d-galactopyranoside (CPRG; Roche Applied Sciences) at 5 mg/ml. The absorbance at 570 nm was read with an ELISA microplate reader (Bio-Rad 680), and values were expressed in OD units/min. Alternatively, luminescence was measured with a Victor3 luminometer (PerkinElmer) and values were expressed in RLU.

### Statistical methods.

Collected data were represented by mean or median values of continuous variables, when appropriate, and as percentages for categorical variables. Comparison of variables between donors harboring HLA-C alleles belonging to the stable/unstable group was performed by χ^2^ test for categorical variables and the *t* test or the Wilcoxon test for continuous variables. Two-way ANOVA was used to ascertain the significance of differences between stable and unstable HLA-C alleles in determining viral infectivity (dependent variable) considering the experimental set as the factor of variability. Two-way ANOVA was used to compare differences between HLA-C*06- and -*07-expressing cells in a controlled cellular model, with the fold change in fluorescence ratio as the dependent variable and the allele (HLA-C*06/HLA-C*07) and the experiment replicas as independent variables. Two-way ANOVA was also used to evaluate the difference in HIV-1 IIIB infectivity in 721.221-CD4 cells with virion infectivity as the dependent variable and the allele (HLA-C*06/HLA-C*07) and the p24 concentration as independent variables. The conventional 5% level of statistical significance was used. Data were analyzed with StataMP 14.0 (Stata Corp., College Station, TX, USA).
