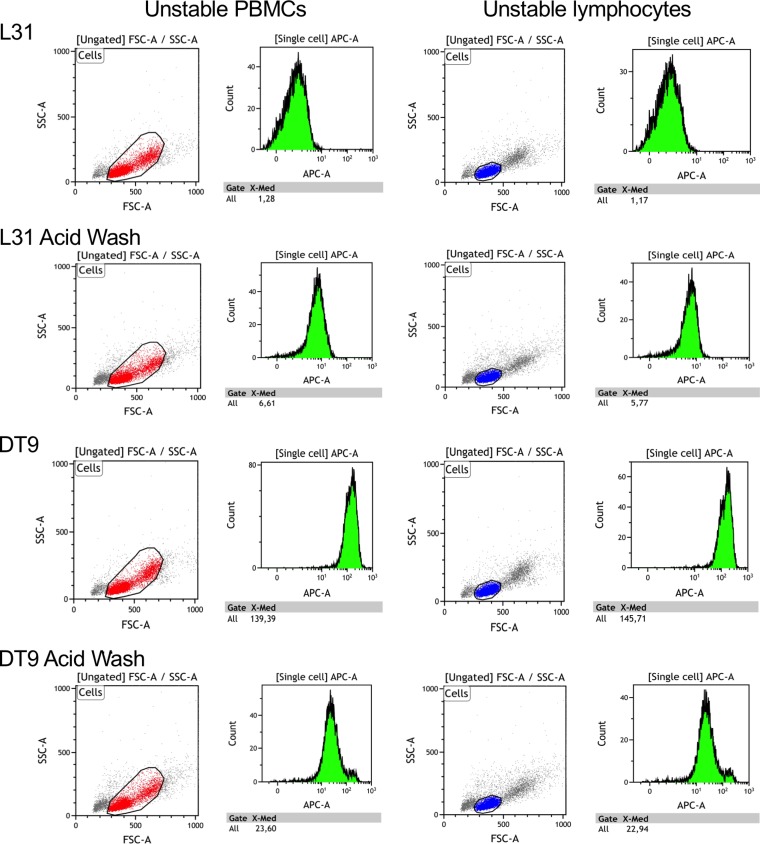# Correction for Parolini et al., “Stability and Expression Levels of HLA-C on the Cell Membrane Modulate HIV-1 Infectivity”

**DOI:** 10.1128/JVI.00911-18

**Published:** 2018-08-16

**Authors:** Francesca Parolini, Priscilla Biswas, Michela Serena, Francesca Sironi, Valentina Muraro, Elisabetta Guizzardi, Lucia Cazzoletti, Maria Teresa Scupoli, Davide Gibellini, Elisabetta Ugolotti, Roberto Biassoni, Alberto Beretta, Mauro Malnati, Maria Grazia Romanelli, Donato Zipeto

**Affiliations:** a Department of Neurosciences, Biomedicine and Movement Sciences, University of Verona, Verona, Italy; b Division of Immunology, Transplantation and Infectious Diseases, IRCCS Ospedale San Raffaele, Milan, Italy; c Service of Transfusion Medicine, AOUI, Verona, Italy; d Department of Diagnostics and Public Health, University of Verona, Verona, Italy; e University Laboratory of Medical Research, Verona, Italy; f IRCCS Institute Giannina Gaslini, Genoa, Italy

## AUTHOR CORRECTION

Volume 92, no. 1, e01711-17, 2018, https://doi.org/10.1128/JVI.01711-17. Page 5: The 16 graphs shown in Fig. 1A (8 histograms and 8 dot plots), referring to the subject with unstable HLA-C alleles, were inadvertent duplicates of the ones in panel B, referring to the subject with stable HLA-C variants. This error occurred during the assembly of the final figure.

The graph portion of Fig. 1A should appear as shown on the following page.

There are no changes to the conclusions of the paper.

**Figure:**